# A Gull Alpha Power Weibull distribution with applications to real and simulated data

**DOI:** 10.1371/journal.pone.0233080

**Published:** 2020-06-12

**Authors:** Muhammad Ijaz, Syed Muhammad Asim, Alamgir, Muhammad Farooq, Sajjad Ahmad Khan, Sadaf Manzoor

**Affiliations:** 1 Department of Statistics, University of Peshawar, Peshawar, KPK, Pakistan; 2 Department of Statistics, Islamia College Peshawar, KPK, Pakistan; Tongii University, CHINA

## Abstract

In this paper, we produced a new family of distribution called Gull Alpha Power Family of distributions (GAPF). A Special case of GAPF is derived by considering the Weibull distribution as a baseline distribution called Gull Alpha Power Weibull distribution (GAPW). The suitability of the proposed distribution derives from its ability to model both the monotonic and non-monotonic hazard rate functions which are a common practice in survival analysis and reliability engineering. Various statistical properties were derived in addition to their special cases. The unknown parameters of the model are estimated using the maximum likelihood method. Moreover, the usefulness of the proposed distribution is supported by using two real lifetime data sets as well as simulated data.

## Introduction

From last few years, researchers made a contribution to the theory of probability so as to remove some of the limitations of the existing probability distributions. For example, the Exponential distribution fails to model the monotonic and non-monotonic hazard rate functions, it can only model the constant hazard rate of an object; Gamma distribution can only model the data with monotonically increasing failure rate. But in practice, various data exists which follows a non-monotonic hazard rate function, for example, the lifetime of an electronic device or the accident rate follows a non-monotonic hazard rate function.

It is usual practice to modify the existing probability models so as to model both the monotonic and non-monotonic hazard rate function and also to provide a suitable fit. One such modification is to produce a generator and then applied to the existing models so as to derive a new probability model. For example, Al-Aqtash et.al [1] produced a new family of distributions using the logit function and derived the special case named as Gumbel-Weibull distribution. Alzaatreh et.al [2] investigated the gamma-X family of distribution and explored the special case by employing the normal distribution as a baseline distribution. Abid, & Abdulrazak [3] Presented a truncated Frechet-G family of distribution. Korkmaz & Genç [4] defined a generalized two-sided class of probability distribution. Alzaghal et.al [5] worked on the T-X family of distributions. Aldeni et.al [6] explained a new family using the quantile functions of the generalized lambda distribution. For other generators, we refer to see [7–10]. The more recent modified Weibull distributions are [3], [4], [6–7], [10].

The scope of developing a new family of distribution is to produce a new probability distribution so as to remove some of the difficulties found in the existing probability distributions. The proposed distribution will not only model the monotonic and non-monotonic hazard rate function, but also increase flexibility and provided a better fit as compared to other probability distribution distributions provided in the literature.

In this paper, a new family of distribution is proposed called Gull Alpha Power Family of distribution. The special case of this family is derived by employing the Weibull distribution called Gull Alpha Power Weibull distribution (GAPW). GAPW is a modified form of the Weibull distribution which can model the non-monotonic hazard rate shapes. Various statistical properties have been derived such as hazard rate function, survival function, and moments. Two real data sets and simulated data are used to check the versatility of the proposed model. The paper detailed discussion is as follows.

## Gull Alpha Power Family (GAPF) of distributions

This section illustrates a new family of probability distributions called Gull Alpha Power Family of distributions or in short GAPF. Let Y is a continuous random variable then the cumulative distribution function (CDF) of Gull Alpha Power Family of distribution is defined as

$$F_{GAPF}(y)=\{\begin{array}{l} \frac{\alpha F(y)}{\alpha^{F(y)}};if\;\alpha >1\\ F(y)=F(y);\;if\;\alpha =1 \end{array}$$ (1)

Where *α* is being the shape parameter and not be zero. The probability density function related to the above CDF takes the form

$$f_{GAPF}(y)=\{\begin{array}{l} \log(\alpha )\alpha^{1-F(y)}(-f(y)F(y))+f(y)\alpha^{1-F(y)};if\;\alpha >1\\ f(y)=f(y);\;if\;\alpha =1 \end{array}$$ (2)

### Gull Alpha Power Weibull distribution (GAPW)

This section illustrates the special form of GAPF by employing the CDF of the Weibull distribution. The CDF of the Weibull distribution [11] is given by

$$F_{W}\left(y\right)=1-e^{-\beta y^{\gamma }},\;y>0$$ (3)

where *β* is the scale parameter and *γ* is the shape parameter.

The Weibull distribution [11] is one of the most important and has been widely used in many real-world problems. For example, in reliability applications, Keshavan et.al [12] used the Weibull distribution to analyze the fracture strength of glass data, Fok et.al [13] utilized to model the failure of brittle materials data, Li et.al [14] by working the failure probability of concrete components. In Geophysics, Al-Hasan & Nigmatullin [15] modeled the wind speed data, to model the data related to earthquake [16] considered the Weibull distribution, and the data concerned with environmental radioactivity was analyzed by Dahm et.al [17]. Tsumoto & Okiai [18] applied the Weibull distribution to the impulse breakdown of oil-filled cable data.

The Weibull distribution can model only increasing, decreasing or a constant failure rate. This distribution fails to model a non-monotonic hazard rate function, for example, a failure rate of the electronic device, accident rate, and infant mortality rate. To achieve this goal, researchers have been constructing different modified versions of the Weibull distribution, for example, Almalki & Yuan [19] presented the New modified Weibull distribution, A new extension of Weibull distribution is presented by [20], a four parameter Weibull distribution is studied by Lemonte et.al [21], Gumbel-Weibull distribution was explored by Al-Aqtash et.al [22], Almheidat et.al [23] investigated the generalized form of the Weibull distribution, Almalki & Nadarajah [24] by working with the discrete form of the Weibull distribution, and a flexible Weibull extension was introduced by Bebbington et.al [25].

Let Y is a continuous random variable which follows GAPW distribution then the CDF and PDF are respectively given by

$$F(y)=\frac{\alpha \left(1-e^{-\beta y^{\gamma }}\right)}{\alpha^{1-e^{-\beta y^{\gamma }}}};\;y>0\;\&\;\alpha ,\beta ,\gamma >0$$ (4)

$$f(y)=\alpha^{e^{-\beta y^{\gamma }}}\beta \gamma y^{\gamma -1}e^{-\beta y^{\gamma }}-\alpha^{e^{-\beta y^{\gamma }}}\log(\alpha )\beta \gamma y^{\gamma -1}\left(1-e^{-\beta y^{\gamma }}\right)e^{-\beta y^{\gamma }}$$ (5)

The proposed distribution contains three parameters that is *β*>0 is the scale and *α*>0, *γ*>0 being the shape parameters.

**Fig 1:**
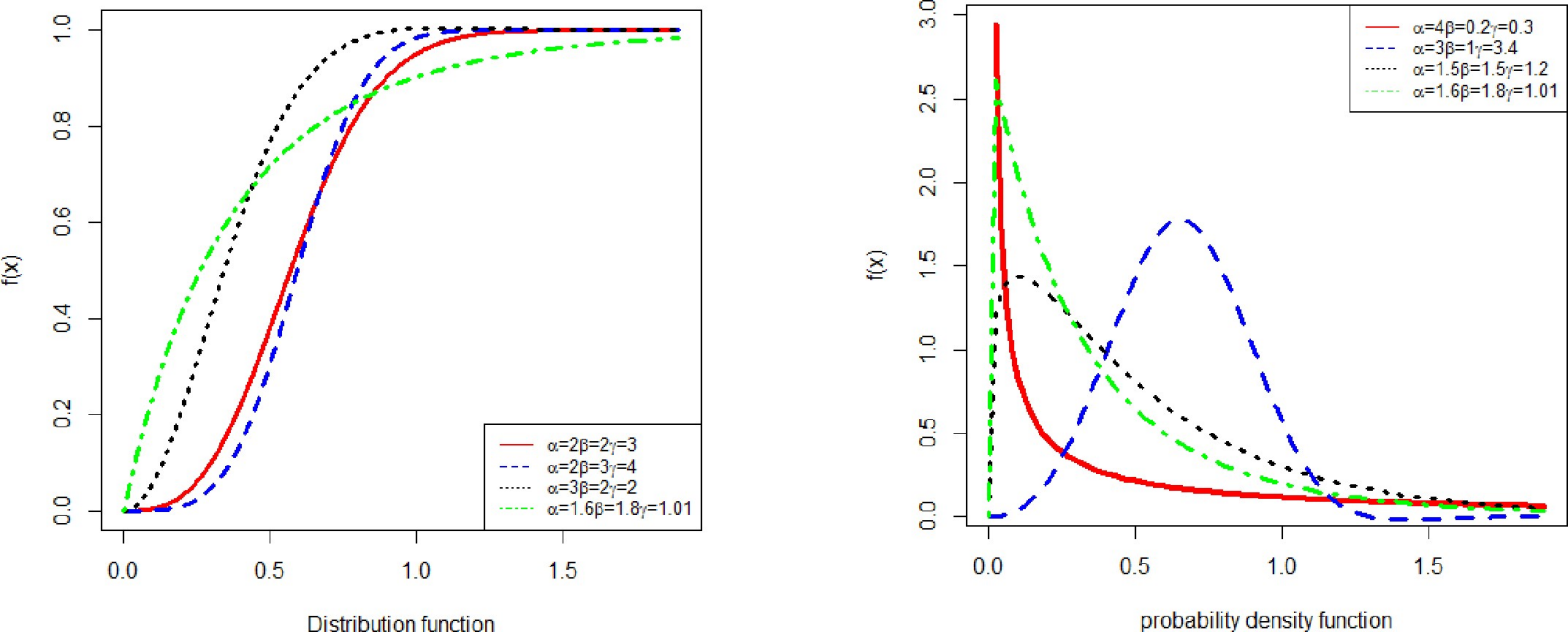
The Pdf and Cdf of GAPW.

The graphical representations of the probability density function and cumulative distribution function with different values of parameters are given in Fig 1.

### The survival and hazard rate function

Let a random variable *Y* follows *GAPW* (*α*,*β*,*γ*). Then the survival function of GAPW is defined as

S(y)=1−F(y),usingexpression(4),weget

$$S(y)=1-\frac{\alpha \left(1-e^{-\beta y^{\gamma }}\right)}{\alpha^{1-e^{-\beta y^{\gamma }}}}=\frac{\alpha^{1-e^{-\beta y^{\gamma }}}-\alpha +\alpha e^{-\beta y^{\gamma }}}{\alpha^{1-e^{-\beta y^{\gamma }}}}$$

$$S(y)=1-\alpha^{e^{-\beta y^{\gamma }}}\left(1-e^{-\beta y^{\gamma }}\right)$$ (6)

Similarly, the failure or hazard rate function of GAPW is defined as $h_{GAPW}(y)=\frac{f(y)}{1-F(y)}$, transforming Eqs (4) and (5), we obtain the result given below

$$=\frac{\alpha^{e^{-\beta y^{\gamma }}}\beta \gamma y^{\gamma -1}e^{-\beta y^{\gamma }}-\alpha^{e^{-\beta y^{\gamma }}}\log(\alpha )\beta \gamma y^{\gamma -1}\left(1-e^{-\beta y^{\gamma }}\right)e^{-\beta y^{\gamma }}}{1-\alpha^{e^{-\beta y^{\gamma }}}\left(1-e^{-\beta y^{\gamma }}\right)}$$

$$=\alpha^{e^{-\beta y^{\gamma }}}\beta \gamma y^{\gamma -1}e^{-\beta y^{\gamma }}\left[1-\frac{\log(\alpha )\left(1-e^{-\beta y^{\gamma }}\right)}{1-\alpha^{e^{-\beta y^{\gamma }}}\left(1-e^{-\beta y^{\gamma }}\right)}\right]$$

$$h_{GAPW}(y)=\alpha^{e^{-\beta y^{\gamma }}}\beta \gamma y^{\gamma -1}e^{-\beta y^{\gamma }}\left[\frac{1-\alpha^{e^{-\beta y^{\gamma }}}\left(1-e^{-\beta y^{\gamma }}\right)-\log(\alpha )-e^{-\beta y^{\gamma }}\log(\alpha )}{1-\alpha^{e^{-\beta y^{\gamma }}}\left(1-e^{-\beta y^{\gamma }}\right)}\right]$$ (7)

**Fig 2:**
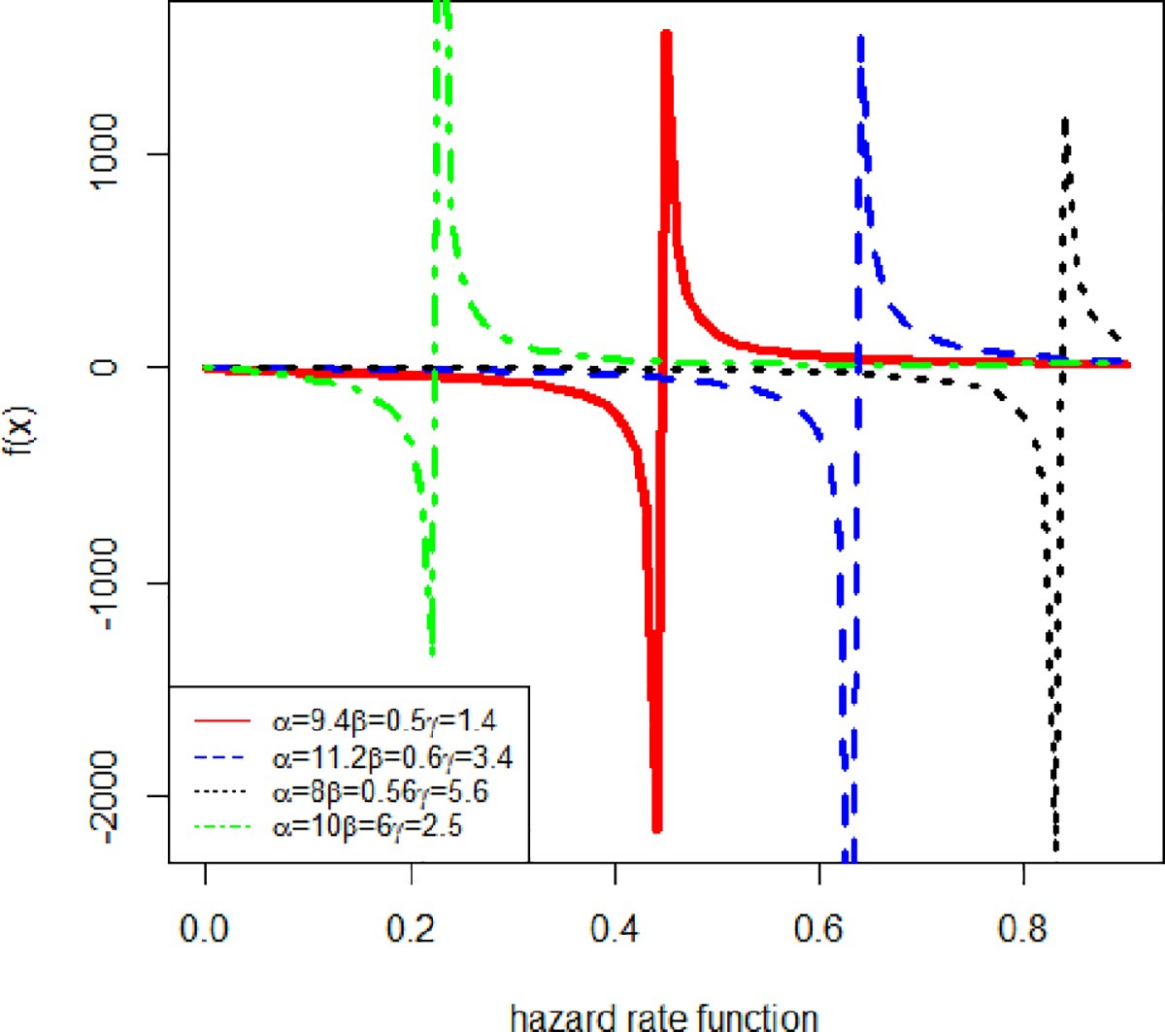
The hazard rate function of GAPW.

Fig 2 shows the behavior of the hazard rate function with different parameter values.

### The quantile function and median

The quantile function is used to conduct a simulation study as well as to measure the median, quartile, octile, decile, and percentile. The quantile function is the real solution of a random variable *Y* to the expression given as

F(y)=μ (8)

Substituting (4), we obtain the result as

$$\frac{\alpha \left(1-e^{-\beta y^{\gamma }}\right)}{\alpha^{1-e^{-\beta y^{\gamma }}}}=\mu ,\;\mathrm{applying}\;\log\;\mathrm{to}\;\mathrm{both}\;\mathrm{sides}\;\mathrm{of}\;\mathrm{the}\;\mathrm{expression}$$

$$\log\left(\left(1-e^{-\beta y^{\gamma }}\right)\alpha^{e^{-\beta y^{\gamma }}}\right)=\log(\mu )$$

The simplified form is then given by

$$\beta y^{\gamma }=\log\left(\frac{\mu }{\frac{1}{\alpha^{e^{\beta y^{\gamma }}}}}\right)$$

Using Mathematica software, we get the following result

$$y={\left(\frac{\mathrm{product}\log\left(\frac{-\log(\alpha )}{\mu }\right)+\log\mu }{\beta }\right)}^{\frac{1}{\gamma }}$$ (9)

where the product Log function W (Z) is defined as

$$W(z)=\frac{\sum_{n=1}^{\infty }{\left(-1\right)}^{n}n^{n-2}}{(n-1)!}z^{n}.$$

For the median, put *u* = 0.5 in Eq (9).

### The r^th^ moments

Let a random variable *Y* has GAPW distribution with parameters *α*,*β*, *and γ* then the r^th^ moments (about the origin), say $u_{r}^{\prime }$ is defined as

$$u_{r}^{\prime }=E\left(y^{r}\right)=\overset{\infty }{\underset{0}{\int }}y^{r}f(y)dy$$ (10)

Recalling (5) we get

$$=\overset{\infty }{\underset{0}{\int }}y^{r}\left(\alpha^{e^{-\beta y^{\gamma }}}\beta \gamma y^{\gamma -1}e^{-\beta y^{\gamma }}-\alpha^{e^{-\beta y^{\gamma }}}\log(\alpha )\beta \gamma y^{\gamma -1}\left(1-e^{-\beta y^{\gamma }}\right)e^{-\beta y^{\gamma }}\right)dy$$ (11)

Solving the first part of the above integral form

$$=\overset{\infty }{\underset{0}{\int }}y^{r}\left(\alpha^{e^{-\beta y^{\gamma }}}\beta \gamma y^{\gamma -1}e^{-\beta y^{\gamma }}\right)dy$$

$$\mathrm{Let}\;z=e^{-\beta y^{\gamma }}\Rightarrow \frac{dz}{dy}=-\beta \gamma y^{\gamma -1}e^{-\beta y^{\gamma }}\Rightarrow -dz=\beta \gamma y^{\gamma -1}e^{-\beta y^{\gamma }}dy,\;\mathrm{and}$$

$$z=e^{-\beta y^{\gamma }}\Rightarrow \log z_{e}=-\beta y^{\gamma }\Rightarrow y=\left(\frac{-\log z}{\beta \gamma }\right);0<y<\infty ;1<z<0.$$

The above integral takes the following form

$$=\overset{0}{\underset{1}{\int }}y^{r}\alpha^{z}(-dz)==\frac{{\left(-1\right)}^{r}}{{\left(\beta \gamma \right)}^{r}}\overset{1}{\underset{0}{\int }}{\left(\log z\right)}^{r}\alpha^{z}dz,$$

Finally, we obtained the result as

$$=-\alpha {\left(-\log(\alpha )\right)}^{-kr-1}\sqrt{kr+1,-\log(\alpha )(z-1)}{|}_{0}^{1}$$ (12)

Now to solve the second part

$$=\overset{\infty }{\underset{0}{\int }}y^{r}\alpha^{e^{-\beta y^{\gamma }}}\log(\alpha )\beta \gamma y^{\gamma -1}\left(1-e^{-\beta y^{\gamma }}\right)e^{-\beta y^{\gamma }}dy$$

$$=\log(\alpha )\overset{\infty }{\underset{0}{\int }}y^{r}\alpha^{e^{-\beta y^{\gamma }}}\beta \gamma y^{\gamma -1}\left(1-e^{-\beta y^{\gamma }}\right)e^{-\beta y^{\gamma }}dy$$

Using the same transformation as earlier, the integral we may write as

$$={\left(-1\right)}^{r}\log\alpha \overset{0}{\underset{1}{\int }}{\left(\frac{\log z}{\beta \gamma }\right)}^{r}\alpha^{z}(1-z)-dz=\frac{{\left(-1\right)}^{r}}{{\left(\beta \gamma \right)}^{r}}\log\alpha \overset{1}{\underset{0}{\int }}{\left(\log z\right)}^{r}\alpha^{z}(1-z)dz$$

We obtained the following result

$$=-\alpha {\left(-\log(\alpha )\right)}^{-kr-2}\sqrt{kr+2,-\log(\alpha )(z-1)}{|}_{0}^{1},\;\mathrm{where};\log(\alpha )\neq 0,kr\neq -3,kr\neq -2$$ (13)

By combining (12) and (13), it’s observed that the r^th^ moment does not exist in general.

### Order statistics

Let *Y*_1_,*Y*_2_,*Y*_3_,…*Y*_*n*_ be ordered random variables from GAPW, then the PDF of the *i*^*th*^ order statistic is given by

$$f_{\left(i;n)(y\right)}=\frac{n!}{(i-1)!(n-i)!}f(y)F{\left(y\right)}^{\left(i-1\right)}{\left[1-F(y)\right]}^{\left(n-i\right)},$$ (14)

Using (4) and (5), the minimum and maximum order statistic of the Gull Alpha Power distribution are given by

$$f_{\left(1:n\right)}(y)=n\left(\alpha^{e^{-\beta y^{\gamma }}}\beta \gamma y^{\gamma -1}e^{-\beta y^{\gamma }}-\alpha^{e^{-\beta y^{\gamma }}}\log(\alpha )\beta \gamma y^{\gamma -1}\left(1-e^{-\beta y^{\gamma }}\right)e^{-\beta y^{\gamma }}\right){\left(1-\frac{\alpha (1-e^{-\beta y^{\gamma }})}{\alpha^{1-e^{-\beta y^{\gamma }}}}\right)}^{n-1}$$

$$f_{\left(1:n\right)}(y)=n(\alpha^{e^{-\beta y^{\gamma }}}\beta \gamma y^{\gamma -1}e^{-\beta y^{\gamma }}-\alpha^{e^{-\beta y^{\gamma }}}\log(\alpha )\beta \gamma y^{\gamma -1}\left(1-e^{-\beta y^{\gamma }}\right)e^{-\beta y^{\gamma }}){\left(\frac{\alpha \left(1-e^{-\beta y^{\gamma }}\right)}{\alpha^{1-e^{-\beta y^{\gamma }}}}\right)}^{n-1}$$

### Parameter estimation

Since the parameters of the probability model are unknown and it is to be estimated using information obtained from a sample. For a detailed discussion on maximum likelihood estimation, we refer to see [26–28]. In this section, the usual method maximum likelihood estimates are used to find out the estimate of the parameters. Let suppose an independent random sample of size n that is *Y*_1,2_*Y*,*Y*_3_…*Y*_*n*_ is selected from GAPW (*α*,*β*,*γ*). The Likelihood function is defined as

$$L=\overset{n}{\underset{i=1}{\prod }}f(y;\alpha ,\beta ,\gamma ),\;\mathrm{Where}\;\alpha ,\beta ,and\;\gamma >0$$

Substituting (5) in the above expression, we get

$$L=\overset{n}{\underset{i=1}{\prod }}\left(\alpha^{e^{-\beta y^{\gamma }}}\beta \gamma y^{\gamma -1}e^{-\beta y^{\gamma }}-\alpha^{e^{-\beta y^{\gamma }}}\log(\alpha )\beta \gamma y^{\gamma -1}\left(1-e^{-\beta y^{\gamma }}\right)e^{-\beta y^{\gamma }}\right)$$

$$=\alpha^{e^{-\beta }\sum y_{i}^{\gamma }}{\left(\beta \gamma \right)}^{n}{\left(\sum y_{i}\right)}^{\gamma -1}e^{-\beta \sum y_{i}^{\gamma }}-\alpha^{e^{-\beta }\sum y_{i}^{\gamma }}\log(\alpha ){\left(\beta \gamma \right)}^{n}{\left(\sum y_{i}\right)}^{\gamma -1}\left(1-e^{-\beta \sum y_{i}^{\gamma }}\right)e^{-\beta \sum y_{i}^{\gamma }}$$

$$\begin{array}{c} \log(L)=e^{-\beta \sum y_{i}^{\gamma }}\log\alpha +n\log(\beta \gamma )+(\gamma -1)\log\left(\sum y_{i}\right)-\beta \sum y_{i}^{\gamma }-e^{-\beta \sum y_{i}^{\gamma }}\log(\alpha )+\log(\log(\alpha ))\\ +n\log(\beta \gamma )+(\gamma -1)\log\left(\sum y_{i}\right)+\log\left(1-e^{-\beta \sum y_{i}^{\gamma }}\right)-\beta \sum y_{i}^{\gamma } \end{array}$$

$$\log L=2n\log(\beta \gamma )+2(\gamma -1)\log\left(\sum y_{i}\right)-2\beta \sum y_{i}^{\gamma }+\log(\log(\alpha ))+\log\left(1-e^{-\beta \sum y_{i}^{\gamma }}\right)$$ (15)

To derive the estimate of the parameters, we have to take the partial derivatives with respect to *α*,*β*,*γ* and then equate the result to zero

$$\frac{d\log L}{d\alpha }=\frac{1}{\log\alpha }\cdot \frac{d(\log(\alpha ))}{d\alpha }=\frac{1}{\log\alpha }\cdot \frac{1}{\alpha }=\frac{1}{\alpha \log\alpha }$$ (16)

$$\frac{d\log L}{d\beta }=\frac{2n\gamma }{\beta \gamma }-2\sum y_{i}^{\gamma }+\frac{(-1)\sum y_{i}^{\gamma }}{\left(1-e^{-\beta \sum \gamma_{i}^{r}}\right)}=\frac{2n}{\beta }-2\sum y_{i}^{\gamma }-\frac{\sum y_{i}^{\gamma }}{\left(1-e^{-\beta \sum y_{i}^{\gamma }}\right)}$$ (17)

$$\frac{d\log L}{d\gamma }=\frac{2n\beta }{\beta \gamma }+2\log\sum y_{i}-2\beta \gamma \sum y_{i}^{\gamma -1}+\frac{e^{-\beta \sum y_{i}^{\gamma }}\beta \gamma \sum y_{i}^{\gamma -1}}{\left(1-e^{-\beta \sum y_{i}^{\gamma }}\right)}$$ (18)

Eqs (16)–(18) is not in closed form. Hence it is difficult to calculate the values of the parameters. However, one can use the iteration procedure used in mathematics that is the Bisection and Newton Raphson method to get the MLE.

### Renyi entropy

By definition, the Renyi entropy of the random variable *Y* belong to GAPW (*α*,*β*,*γ*) is given by

$$R_{H}(y)=\frac{1}{1-p}\log\int_{0}^{\infty }f^{p}(y)dy$$ (19)

$$\mathrm{Where}\;f(y)=\alpha^{e^{-\beta y^{\gamma }}}\beta \gamma y^{\gamma -1}e^{-\beta y^{\gamma }}-\alpha^{e^{-\beta y^{\gamma }}}\log(\alpha )\beta \gamma y^{\gamma -1}\left(1-e^{-\beta y^{\gamma }}\right)e^{-\beta y^{\gamma }}$$

$$\overset{\infty }{\underset{0}{\int }}f^{p}(y)dy=\overset{\infty }{\underset{0}{\int }}{\left[\alpha^{e^{-\beta y^{\gamma }}}\beta \gamma y^{\gamma -1}e^{-\beta y^{\gamma }}-\alpha^{-\beta y^{\gamma }}\log(\alpha )\beta \gamma y^{\gamma -1}\left(1-e^{-\beta y^{\gamma }}\right)e^{-\beta y^{\gamma }}\right]}^{p}dy$$

$$=\overset{\infty }{\underset{0}{\int }}{\left[\alpha^{e^{-\beta y^{\gamma }}}\beta \gamma y^{\gamma -1}e^{-\beta y^{\gamma }}\right]}^{p}{\left[1-\log(\alpha )\left(1-e^{-\beta y^{\gamma }}\right)\right]}^{p}dy$$ (20)

Using the expression given below

$${\left[1-\log(\alpha )\left(1-e^{-\beta y^{\gamma }}\right)\right]}^{p}=\sum_{k=j=0}^{\infty }\log{\left(\alpha \right)}^{k}{\left(-1\right)}^{k+j}\left({}^{p}C_{k}\right){(}^{k}C_{j}){\left(e^{-\beta y^{\gamma }}\right)}^{j}$$

Hence (20) takes the following form

$$={\left(\beta \gamma \right)}^{p}\overset{\infty }{\underset{0}{\int }}{\left[\alpha^{e^{-\beta y^{\gamma }}}y^{\gamma -1}e^{-\beta y^{\gamma }}\right]}^{p}\overset{\infty }{\underset{k=j=0}{\sum }}\log{\left(\alpha \right)}^{k}{\left(-1\right)}^{k+j}{(}^{p}C_{k}{)(}^{k}C_{j}){\left(e^{-\beta y^{\gamma }}\right)}^{j}dy$$

$$={\left(\beta \gamma \right)}^{p}\overset{\infty }{\underset{k=j=0}{\sum }}\log{\left(\alpha \right)}^{k}{\left(-1\right)}^{k+j}{(}^{p}C_{k}{)(}^{k}C_{j})\overset{\infty }{\underset{0}{\int }}{\left[\alpha^{e^{-\beta y^{\gamma }}}y^{\gamma -1}e^{-\beta y^{\gamma }}\right]}^{p}{\left(e^{-\beta y^{\gamma }}\right)}^{j}dy$$ (21)

by solving the above expression, we get

$$={\left(\beta \gamma \right)}^{p}\overset{\infty }{\underset{k=j=0}{\sum }}\log{\left(\alpha \right)}^{k}{\left(-1\right)}^{k+j}{(}^{p}C_{k}){(}^{k}C_{j})\overset{\infty }{\underset{n=m=0}{\sum }}\frac{{\left(p\log(\alpha )\right)}^{n}}{n!}\frac{{\left(-\beta (p+j+n)y^{\gamma }\right)}^{m}}{m!}\frac{y^{p(\gamma -1)+m\gamma +1}}{p(\gamma -1)+m\gamma +1}{|}_{0}^{\infty }$$ (22)

Replace (22) in (19), we obtained the following result for the Renyi entropy

$$R_{H}(y)=\frac{1}{1-p}\log\left(\begin{array}{l} {\left(\beta \gamma \right)}^{p}\sum_{k=j=0}^{\infty }\log{\left(\alpha \right)}^{k}{\left(-1\right)}^{k+j}{(}^{p}C_{k}){(}^{k}C_{j})\sum_{n=m=0}^{\infty }\frac{{\left(p\log(\alpha )\right)}^{n}}{n!}\\ {\frac{{\left(-\beta (p+j+n)y^{\gamma }\right)}^{m}}{m!}\frac{y^{p(\gamma -1)+m\gamma +1}}{p(\gamma -1)+m\gamma +1}|}_{0}^{\infty } \end{array}\right)$$ (23)

### Mode

The mode of the random variable *Y* is defined by the following equation.

$$f^{\prime }(y)=0$$

$$\frac{df(y)}{dy}=\frac{d}{dy}\left(\alpha^{e^{-\beta y^{\gamma }}}\beta \gamma y^{\gamma -1}e^{-\beta y^{\gamma }}-\alpha^{e^{-\beta y^{\gamma }}}\log(\alpha )\beta \gamma y^{\gamma -1}\left(1-e^{-\beta y^{\gamma }}\right)e^{-\beta y^{\gamma }}\right)$$

$$=-\log(\alpha )\beta \gamma \frac{d}{dy}\left(\alpha^{e^{-\beta y^{\gamma }}}y^{\gamma -1}\left(1-e^{-\beta y^{\gamma }}\right)e^{-\beta y^{\gamma }}\right)+\beta \gamma \frac{d}{dy}\left(\alpha^{e^{-\beta y^{\gamma }}}y^{\gamma -1}e^{-\beta y^{\gamma }}\right)$$

$$=\alpha^{e^{-\beta y^{\gamma }}}\beta \gamma y^{\gamma -2}e^{-3\beta y^{\gamma }}\left[\begin{array}{l} \left\{(\log\alpha -1)\beta \gamma y^{\gamma }+(\gamma -\log(\alpha )\gamma )+\log(\alpha )-1\right\}e^{2\beta y^{\gamma }}\\ +\left\{\left(\log^{2}(\alpha )-\beta \log(\alpha )\right)\beta \gamma y^{\gamma }+\log(\alpha )\gamma -\log(\alpha )\right\}e^{\beta y^{\gamma }}-\log^{2}(\alpha )\beta \gamma y^{\gamma } \end{array}\right]$$

The more simplified form of the above expression may be written as

$$\alpha^{e^{-\beta y^{\gamma }}}\beta \gamma y^{\gamma -2}e^{-3\beta y^{\gamma }}=0$$ (24)

Using Mathematica software, it has been observed that the density function will be maximum only at *y* = 0

### Skewness and kurtosis

The mathematical form of the Galton Skewness and Moors kurtosis of the GAPW distribution with three parameters are defined by the following relationship

$$S_{K}=\frac{Q(3/4)+Q(1/4)-2Q(2/4)}{Q(3/4)-Q(1/4)}$$ (25)

$$K_{M}=\frac{Q(7/8)+Q(3/8)-Q(5/8)-Q(1/8)}{Q(3/4)-Q(1/4)}$$ (26)

Where *Q* describe different quartiles values. Table 1 illustrates the numerical description of the Skewness and Kurtosis for different values of parameters.

**Table 1:** Numerical values of skewness and kurtosis.

*α*	*β*	*γ*	Skewness	Kurtosis
0.1	0.1	0.1	0.8743687	2.258266
0.1	0.2	0.3	0.2625882	0.9188042
0.1	0.4	0.5	0.008697689	0.9147886
0.1	0.6	0.6	-0.059749	0.9656408
0.2	0.3	0.1	0.9696736	3.511172
0.3	0.3	0.2	0.916672	2.373731
0.4	0.3	0.2	0.7812134	9.273318
0.8	0.5	0.6	-0.2128008	1.200246
0.9	0.6	1	-0.2271696	1.220157
1	1	1	-0.2618595	1.30627

### Mean Residual Life (MRL)

The mean residual life of the gull alpha power distribution with parameters *α*,*β*,*γ* is define as

$$MRL_{GAPW}(y)=E(Y-y/Y>y)=\frac{1}{S(Y;\alpha ,\beta ,\gamma )}\overset{\infty }{\underset{\gamma }{\int }}f(t;\alpha ,\beta ,\gamma )dt-y$$ (27)

$$\mathrm{where},\;S(Y;\alpha ,\beta ,\gamma )=1-\alpha^{e^{-\beta y^{\gamma }}}(1-e^{-\beta y^{\gamma }})\;\mathrm{and}$$

$$f(t;\alpha ,\beta ,\gamma )=\alpha^{e^{-\beta t^{\gamma }}}\beta \gamma t^{\gamma -1}e^{-\beta t^{\gamma }}-\alpha^{e^{-\beta t^{\gamma }}}\log(\alpha )\beta \gamma t^{\gamma -1}\left(1-e^{-\beta t^{\gamma }}\right)e^{-\beta t^{\gamma }}$$

Plugging in the above two expressions in (27), we get

$$=\frac{1}{1-\alpha^{e^{-\beta y^{\gamma }}}\left(1-e^{-\beta y^{\gamma }}\right)}\left[\overset{\infty }{\underset{y}{\int }}\alpha^{e^{-\beta t^{\gamma }}}\beta \gamma t^{\gamma -1}e^{-\beta t^{\gamma }}dt-\overset{\infty }{\underset{y}{\int }}\alpha^{e^{-\beta t^{\gamma }}}\log(\alpha )\beta \gamma t^{\gamma -1}\left(1-e^{-\beta t^{\gamma }}\right)e^{-\beta t^{\gamma }}dt\right]-y$$ (28)

By solving (28) finally, we obtained the following result

$$=\frac{1}{1-\alpha^{e^{-\beta y^{\gamma }}}\left(1-e^{-\beta y^{\gamma }}\right)}\left[\left(\frac{\alpha^{e^{-\beta y^{\gamma }}}}{\log(\alpha )}-\frac{1}{\log(\alpha )}\right)-l\mathrm{og}(\alpha )\left[\frac{\alpha^{e^{-\beta y^{\gamma }}}-1}{\log(\alpha )}-\frac{\left(\log(\alpha )e^{-\beta y^{\gamma }}-1\right)\alpha^{e^{-\beta y\gamma }}}{{\left(\log(\alpha )\right)}^{2}}+\frac{\alpha (\log(\alpha )-\alpha }{{\left(\log(\alpha )\right)}^{2}}\right]\right]-y$$ (29)

### Special cases

This section illustrates two special cases of the Gull Alpha Power Weibull distribution.

- Case. *γ* = 1
  When we put *γ* = 1 in (4) and (5), then it shall be referring to the CDF and PDF of the Gull Alpha Power Exponential distribution (GAPE). The mathematical forms are described as
  $$F(y)=\frac{\alpha \left(1-e^{-\beta y}\right)}{\alpha^{1-e^{-\beta y}}};\;y>0\&\alpha ,\beta >0$$ (30)
  $$f(y)=\alpha^{e^{-\beta y}}\beta e^{-\beta y}-\alpha^{e^{-\beta y}}\log(\alpha )\beta \left(1-e^{-\beta y}\right)e^{-\beta y}$$ (31)
- Case. *γ* = 2
  If we replace *γ* = 2 in the expressions (4) and (5), the derived probability function will stand for the Gull Alpha Power Rayleigh distribution (GAPR). The CDF and PDF of GAPR are respectively given by
  $$F(y)=\frac{\alpha \left(1-e^{-\beta y^{2}}\right)}{\alpha^{1-e^{-\beta y^{2}}}};\;y>0\&\alpha ,\beta >0$$ (32)
  $$f(y)=2\alpha^{e^{-\beta y^{2}}}\beta y^{2-1}e^{-\beta y^{2}}-2\alpha^{e^{-\beta y^{2}}}\log(\alpha )\beta y^{1}\left(1-e^{-\beta y^{2}}\right)e^{-\beta y^{2}}$$ (33)

### Applications

In this section, we provide two real life applications of the proposed model in which one data set follows a non-monotonic hazard rate shape and the second data follows a monotonic hazard rate shape so as to achieve the objectives and delineate efficiency of the proposed model. The performance of the model is judged by commonly used goodness of fit measures including Cramer-von mises (W), Anderson darling (A), Akaike information criteria (AIC), Consistent Akaike information criteria (CAIC), Hannan and quin information criteria (HQIC), and Bayesian information criteria (BIC). The mathematical form of these criteria is defined by

$$A=-n-\frac{1}{n}\sum_{i=1}^{n}\left(2i-1\right)\left[\log F\left(X_{i}\right)+\log\left(1-F\left(X_{n-i+1}\right)\right)\right]$$

$$W={\sum_{i=1}^{n}\left[F\left(X_{i}\right)-\frac{2i-1}{2n}\right]}^{2}+\frac{1}{12n}$$

$$AIC=-2L+2p,\;AICc=AIC+\frac{2p(p+1)}{n-p-1},\;CAIC=-2L+P\left\{\log(n)+1\right\}$$

BIC=Plog(n)−2L,HQIC=−2L+2Plog{log(n)}.

where, $L=L(\hat{\psi };y_{i})$ is the maximized likelihood function and *y*_*i*_ is the given random sample, $\hat{\psi }$ is the maximum likelihood estimator and *p* is the number of parameters in the model.

As a general rule, a probability model with fewer values of these criteria should be considered the best fitted model among other probability distributions.

### Data set 1: Remission time of Bladders cancer patients

The data set consists of the remission time of 128 bladder cancer patients. The data set is taken from Aldeni and Famoye [29] with the values are as follows

0.080, 0.200, 0.400, 0.500, 0.510, 0.810, 0.900, 1.050, 1.190, 1.260, 1.350, 1.400, 1.460, 1.760, 2.020, 2.020, 2.070, 2.090, 2.230, 2.260, 2.460, 2.540, 2.620, 2.640, 2.690, 2.690, 2.750, 2.830, 2.870, 3.020, 3.250, 3.310, 3.360, 3.360, 3.480, 3.520, 3.570, 3.640, 3.700, 3.820, 3.880, 4.180, 4.230, 4.260, 4.330, 4.340, 4.400, 4.500, 4.510, 4.870, 4.980, 5.060, 5.090, 5.170, 5.320, 5.320, 5.340, 5.410, 5.410, 5.490, 5.620, 5.710, 5.850, 6.250, 6.540, 6.760, 6.930, 6.940, 6.970, 7.090, 7.260, 7.280, 7.320, 7.390, 7.590, 7.620, 7.630, 7.660, 7.870, 7.930, 8.260, 8.370, 8.530, 8.650, 8.660, 9.020, 9.220, 9.470, 9.740, 10.06, 10.34, 10.66, 10.75, 11.25, 11.64, 11.79, 11.98, 12.02, 12.03, 12.07, 12.63, 13.11, 13.29, 13.80, 14.24, 14.76, 14.77, 14.83, 15.96, 16.62, 17.12, 17.14, 17.36, 18.10, 19.13, 20.28, 21.73, 22.69, 23.63, 25.74, 25.82, 26.31, 32.15, 34.26, 36.66, 43.01, 46.12, 79.05.

**Fig 3:**
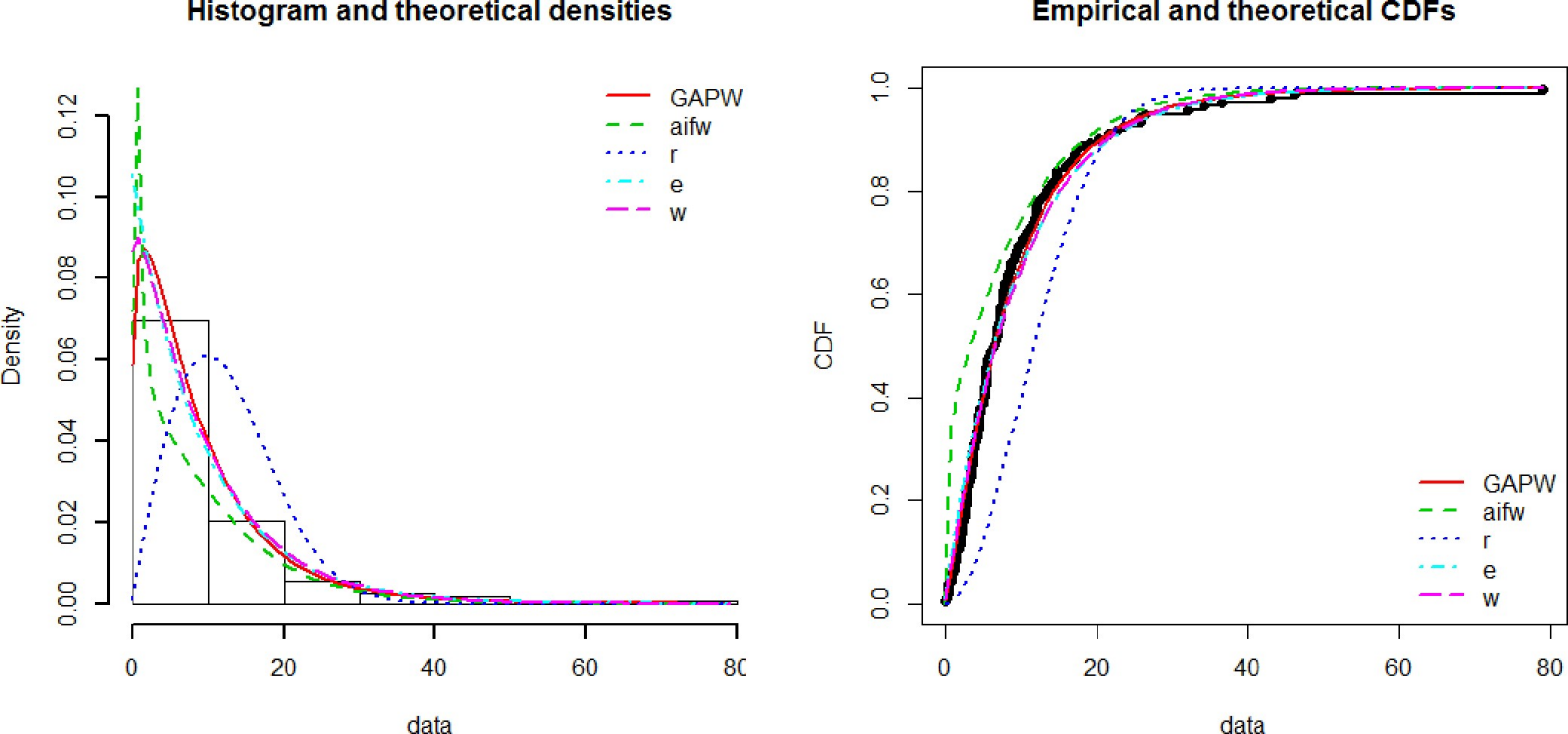
Theoretical and empirical Pdf and Cdf of GAPW.

**Fig 4:**
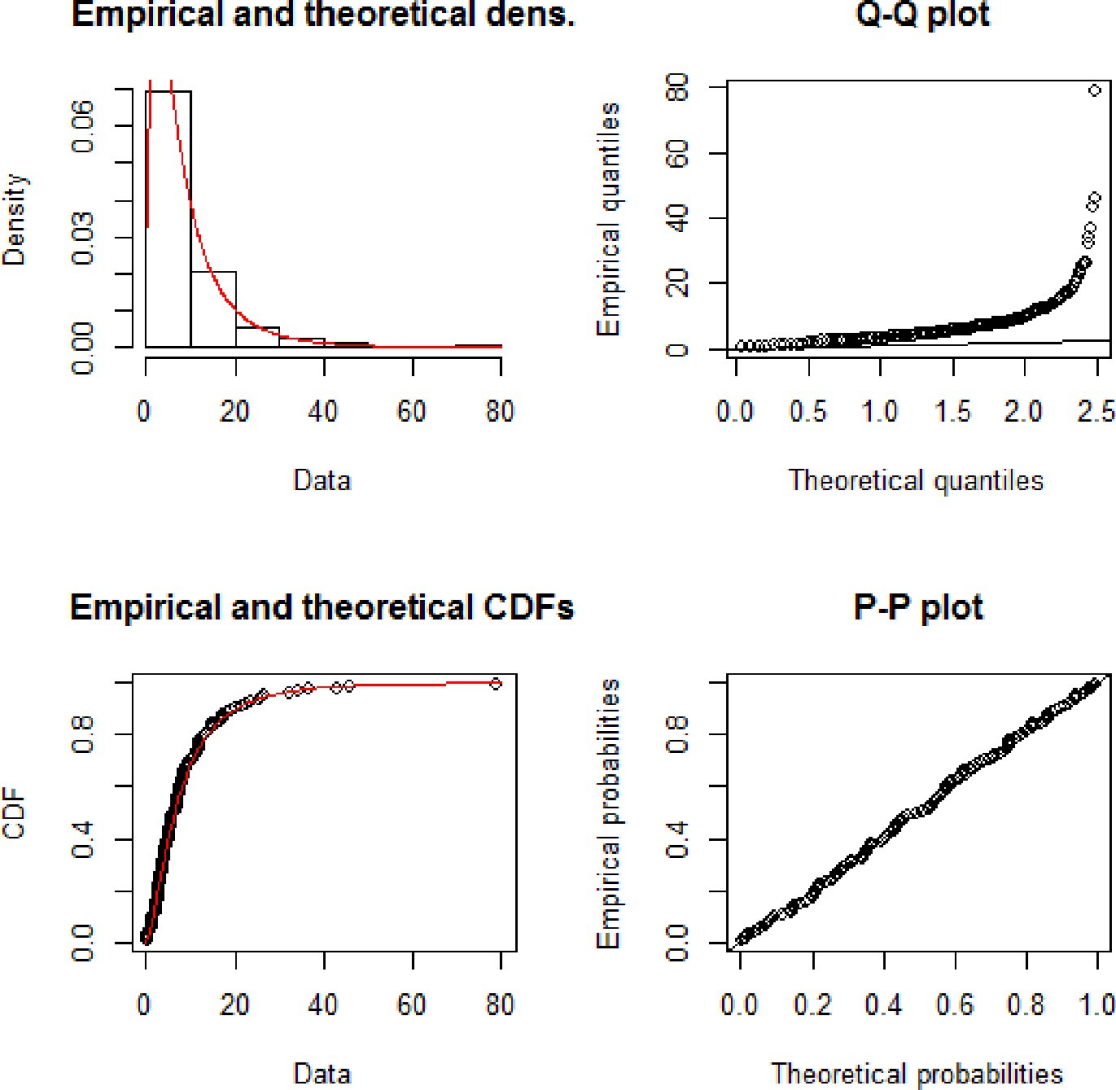
Theoretical and empirical Pdf and Cdf with Q-Q plot and P-P plot for GAPW.

**Fig 5:**
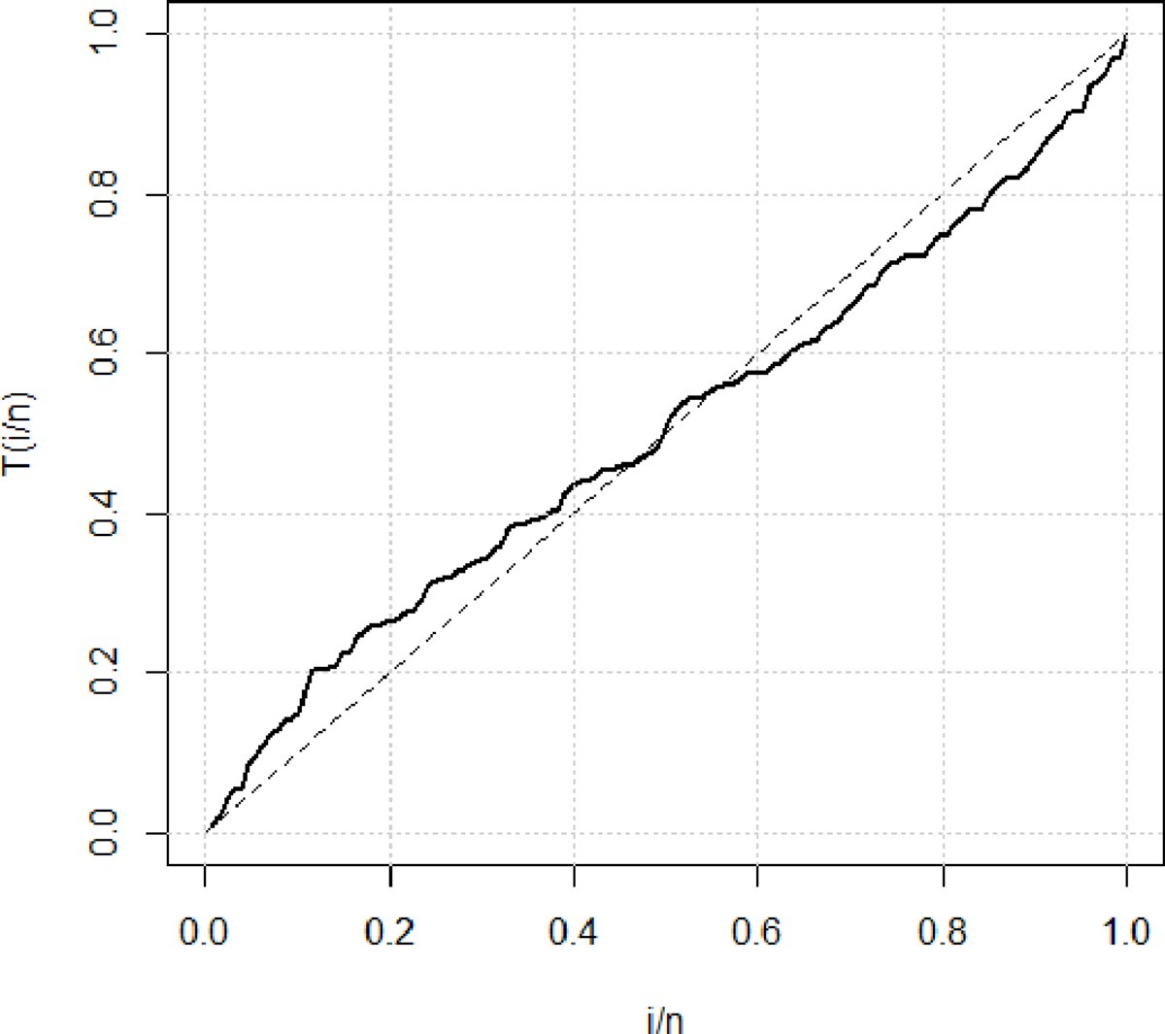
TTT plot of the bladder cancer patient data.

Fig 3 shows the theoretical and empirical pdf and cdf of the GAPW distribution using the bladder cancer patient’s data and it is observed that the GAPW is the best-fitted line as compared to others. Fig 4 demonstrates the Q-Q and P-P plot of the bladder cancer patient data. The TTT plot in Fig 5 clearly shows that this data follows a non-monotonic hazard rate shapes. Table 2 reflects the maximum likelihood estimates, standard errors, and the log-likelihood values. Table 3 defines the goodness of fit measures for the bladder cancer data. It has been observed that the goodness of fit measures has fewer values for GAPW while analyzing the bladder cancer data. Hence the proposed distribution provides a better fit as compared Weibull exponential (W.E), exponential (E), Weibull (W), Rayleigh (R) and Algoharai inverse flexible Weibull (AIFW) distribution.

**Table 2:** Maximum likelihood estimates and their standard errors.

Model	Mle	Standard error	-log(likelihood)
GAPW	0.00590119 0.79751413 0.53355796	0.005280265 0.121546525 0.046158047	
			409.9908

W.E	3.95810505 0.01796843 0.85819193	1.214089581 0.004666546 0.059280045	
			419.8998

W	0.09438292 1.04576466	0.01912624 0.06742473	
			414.0874
Exp	0.1067695	0.009436355	414.3419
Rayleigh	0.005079773	0.0004307331	491.2659
AIFW	0.1677404 0.1231948	0.02508775 0.01045528	451.0704

**Table 3:** Goodness of fit measures of the GAPW for bladder cancer data.

Models	W	A	AIC	CAIC	BIC	HQIC
GAPW	0.02533431	0.1608187	825.9815	826.1751	834.5376	829.4579
W.E	0.2145276	1.282891	845.7996	845.9931	854.3557	849.276
W	0.1308177	0.7832353	832.1747	832.2707	837.8788	834.4923
Exp	0.1192893	0.7159703	830.6838	830.7155	833.5358	831.8426
Rayleigh	0.4669078	2.732901	984.5318	984.5635	987.3838	985.6906
AIFW	0.5735798	3.457475	906.1409	906.2369	911.8449	908.4585

### Data set 2: Bank customers Data

The data set waiting time of 100 bank customers is taken from Ghitany et al. [30]. The data set values are given below

0.8,0.8,1.3,1.5,1.8,1.9,1.9,2.1,2.6,2.7,2.9,3.1,3.2,3.3,3.5,3.6,4,4.1,4.2,4.2,4.3,4.3,4.4,4.4,4.6,4.7,4.7,4.8,4.9,4.9,5.0,5.3,5.5,5.7,5.7,6.1,6.2,6.2,6.2,6.3,6.7,6.9,7.1,7.1,7.1,7.1,7.4,7.6,7.7,8,8.2,8.6,8.6,8.6,8.8,8.8,8.9,8.9,9.5,9.6,9.7,9.8,10.7,10.9,11.0,11.0,11.1,11.2,11.2,11.5,11.9,12.4,12.5,12.9,13.0,13.1,13.3,13.6,13.7,13.9,14.1,15.4,15.4,17.3,17.3,18.1,18.2,18.4,18.9,19.0,19.9,20.6,21.3,21.4,21.9,23,27,31.6,33.1,38.5.

**Fig 6:**
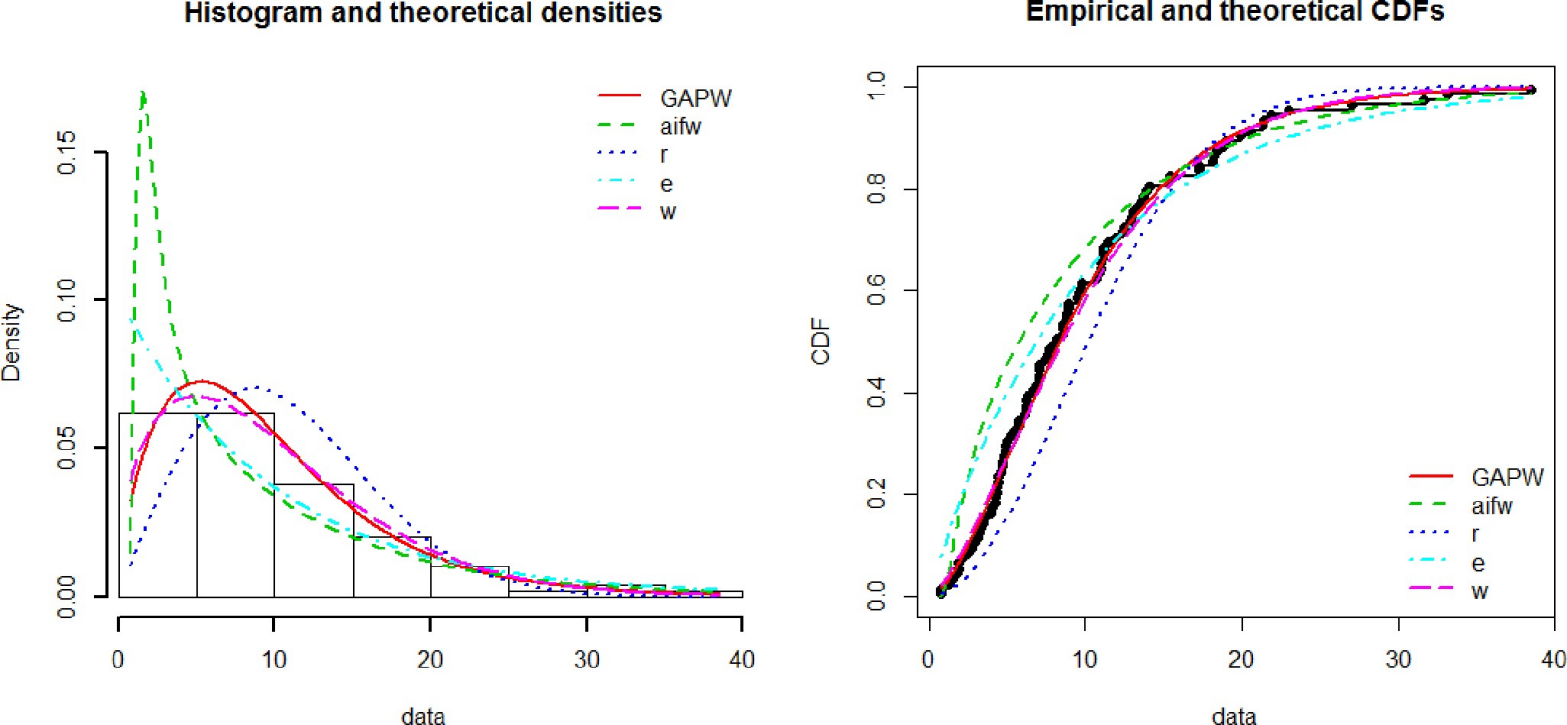
Theoretical and empirical Pdf and Cdf of GAPW.

**Fig 7:**
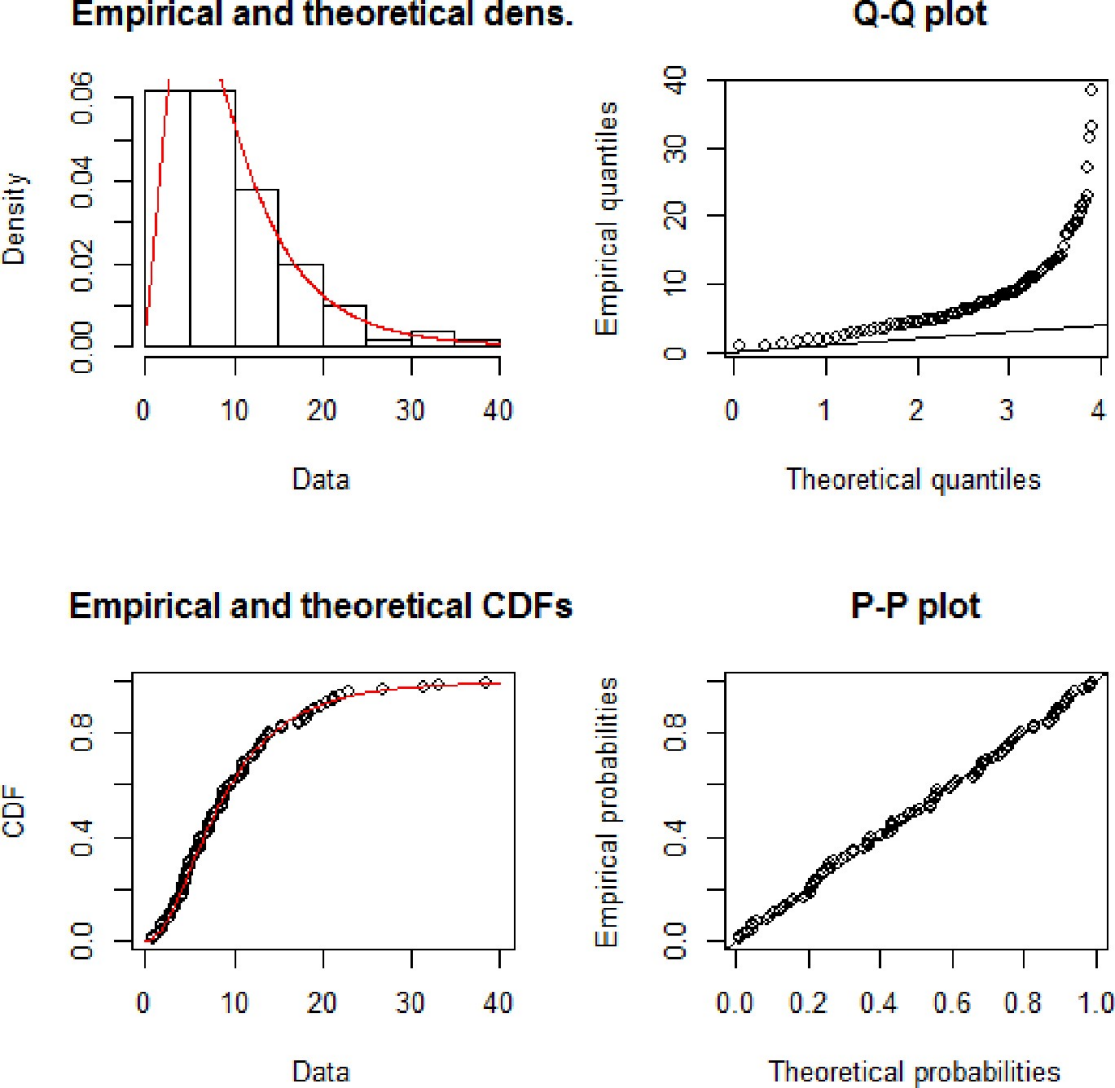
Theoretical and empirical Pdf and Cdf with Q-Q plot and P-P plot for GAPW.

**Fig 8:**
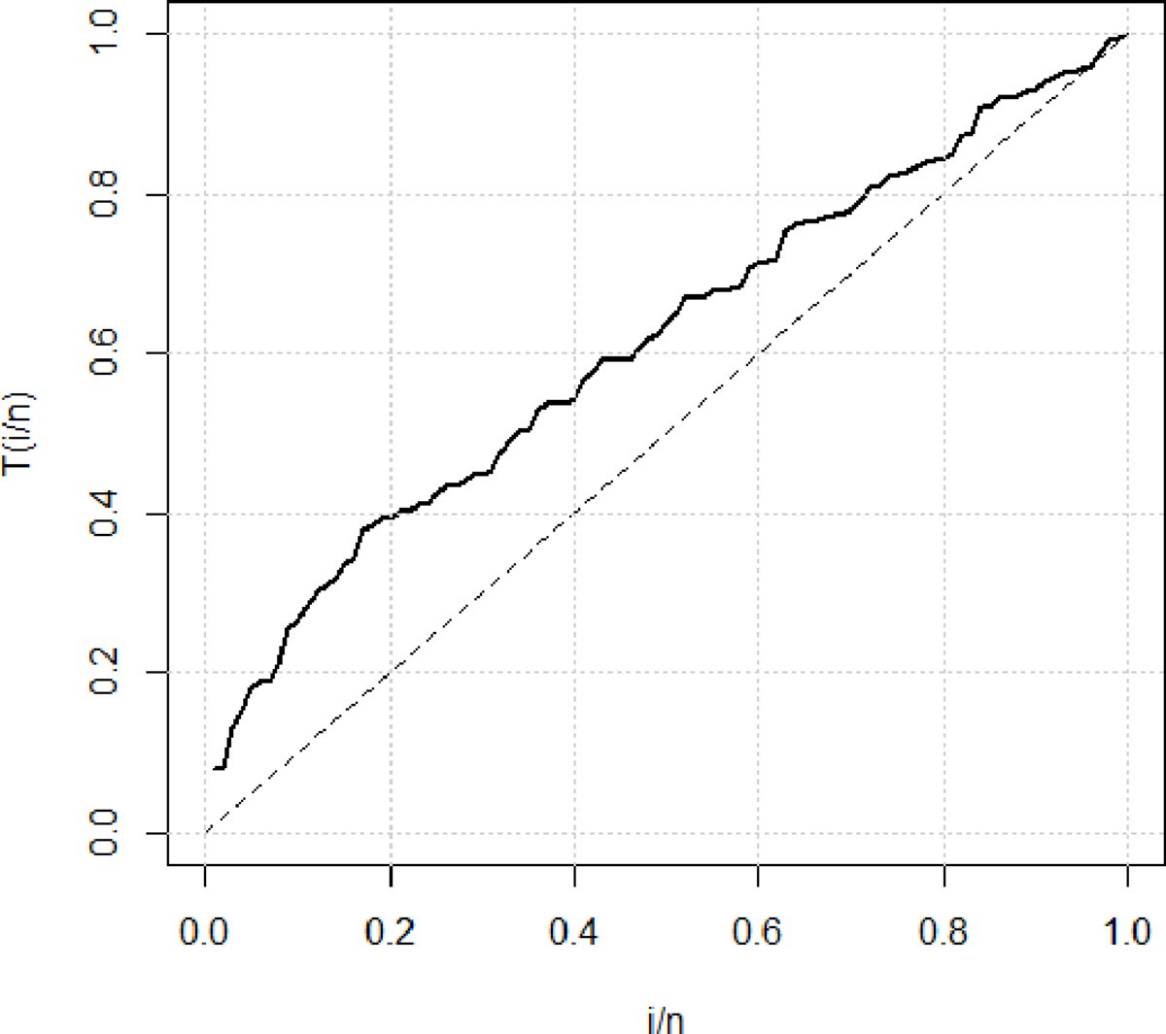
TTT plot of bank customers data.

Fig 6 illustrates the theoretical and empirical Pdf and Cdf of the GAPW distribution using the 100 bank customer’s data. The graph clearly shows that the red line is the best-fitted line to theoretical data. Fig 7 displays the Q-Q and P-P plot of the bank customer’s data. The TTT plot in Fig 8 clearly shows that this data follows a monotonic hazard rate shapes. Table 4 gives the maximum likelihood estimates of the unknown parameters of GAPW including the standard errors, and the log-likelihood values. The values given in Table 5 declared that GAPW leads to a better fit than other versions of the Weibull distribution like Weibull exponential (W.E), exponential (E), Weibull (W), Rayleigh (R) and Algoharai inverse flexible Weibull (AIFW) distribution.

**Table 4:** Maximum likelihood estimates and their standard errors.

Model	Mle	Standard error	-log(likelihood)
GAPW	0.004584114 0.540116500 0.679696285	0.007821438 0.164012222 0.088528859	317.4891
W.E	3.97641055 0.02509669 1.24349114	4.16205665 0.01545783 0.15095850	320.9662
W	0.02971531 1.46144250	0.008598382 0.102215074	318.745
Exp	0.1012424	0.01012326	329.0209
Rayleigh	0.006676124	0.0006522215	329.2404
AIFW	1.6423171 0.1153154	0.17122546 0.01167334	330.7856

**Table 5:** Goodness of fit measures of the GAPW for bank customers data.

Model	W	A	AIC	CAIC	BIC	HQIC
GAPW	0.01939983	0.1355293	640.9783	641.2283	648.7938	644.1413
W.E	0.112468	0.7070021	647.9323	648.1823	655.7479	651.0954
W	0.06265989	0.3945532	641.4899	641.6136	646.7003	643.5986
Exp	0.02703835	0.1790246	660.0418	660.0826	662.6469	661.0961
Rayleigh	0.1265804	0.7863305	660.4807	660.5216	663.0859	661.5351
AIFW	0.1703407	1.219407	665.5711	665.6948	670.7815	667.6798

### Simulations

To perform simulations an expression (9) was used to generate artificial data from the GAPW distribution. The simulations are performed 100 times with a different set of parameters with different sample of size *n*. The maximum likelihood estimates and their standard errors are given in Table 6. The tabulated values clearly show as we increase the sample size, both the ml estimates and the standard errors are decreases. The general formula for computing the mean square error and bias are as follows

$$MSE=\frac{1}{W}\overset{W}{\underset{i=1}{\sum }}{\left(\alpha_{i}-\alpha \right)}^{2}$$

$$Bias=\frac{1}{W}\overset{W}{\underset{i=1}{\sum }}\left(\alpha_{i}-\alpha \right)$$

**Table 6:** Maximum likelihood Estimates and their standard errors.

Actual values		ML Estimate			Standard deviations		
*α β γ*	n	*α β γ*			*α β γ*		
	30	52.356569	23.944706	-1.425906	9.2277595	7.2776554	0.1274975
	50	50.95129	24.95536	-1.43942	6.9704413	6.0708245	0.1017499
0.009, 0.007, 2.5	60	50.664975	26.246471	-1.466574	6.31080601	5.72063698	0.09179273
0.002, 0.03, 2.4	30	48.053781	30.374984	2.557561	8.5074528	11.2457262	0.2579958
	40	54.455222	38.392647	-2.634047	8.3220182	10.0698906	0.1794368
	50	50.95129	24.95536	-1.43942	6.9704413	6.0708245	0.1017499
0.004, 0.04, 2.4	15	43.061076	27.780158	-2.690999	10.4921960	12.5321045	0.3456288
	30	44.815687	32.610212	-2.716042	7.8711814	11.2785370	0.2546263
	60	58.716823	26.033329	-2.651458	7.4268484	6.0626125	0.1738104
0.009, 0.05 3.5	30	74.154700	38.831954	-4.727522	13.5628738	14.8703835	0.4515338
	45	77.262356	35.034728	-4.703549	11.5691089	10.7248896	0.3635661
	60	63.771730	37.754394	-4.728007	8.1322331	10.1954503	0.3217287

## Conclusion

In this paper, we produced a new generator called Gull Alpha Power Family of distributions or in short GAPF. A special case of this family was derived by employing the CDF of the Weibull distribution as a baseline distribution. The special case is known as Gull Alpha Power Weibull distribution (GAPW). Various statistical properties have been discussed in addition to the parameter estimation using the maximum likelihood method. In future, a researcher may conduct a study on estimation of the parameters of the proposed model under Bayesian paradigm by using informative and non-informative priors. For a detailed discussion on Bayesian estimation we refer to see [31–34]. Furthermore, we have explored the special cases of GAPW. The significance of the proposed model is justified by using two real data sets as well as the simulated data. The TTT plot of the bladder cancer patient’s data clearly demonstrates that this data follows a non-monotonic hazard rate shape. While the TTT plot of the bank customers data follows a monotonic hazard rate shape. It has been observed that the proposed model performs well in both the non-monotonic and a monotonic hazard rate shapes as compared to the Weibull (W), Weibull Exponential (W.E), Exponential (E), Rayleigh (R), and Algoharai Inverse Flexible Weibull (AIFW) distribution.

## Supporting information

S1 Table Numerical values of skewness and kurtosis. (DOCX)

S2 Table Maximum likelihood estimates and their standard errors. (DOCX)

S3 Table Goodness of fit measures of the GAPW for bladder cancer data. (DOCX)

S4 Table Maximum likelihood estimates and their standard errors. (DOCX)

S5 Table Goodness of fit measures of the GAPW for bladder cancer data. (DOCX)

S6 Table Maximum likelihood Estimates and their standard errors. (DOCX)

S1 Data Remission time of Bladders cancer patients [26]. (DOCX)

S2 Data Bank customers data [27]. (DOCX)
