# Supplementary material for: A Gull Alpha Power Weibull distribution with applications to real and simulated data
Source: PLoS One. 2020 Jun 12;15(6):e0233080. doi: 10.1371/journal.pone.0233080 (PMC7292407; doi:10.1371/journal.pone.0233080)
Supplement: S1 Table — (DOCX) [file pone.0233080.s001.docx]

**Table 1. Numerical values of skewness and Kurtosis**

|  |  |  | **Skewness** | **Kurtosis** |
| --- | --- | --- | --- | --- |
| 0.1 | 0.1 | 0.1 | 0.8743687 | 2.258266 |
| 0.1 | 0.2 | 0.3 | 0.2625882 | 0.9188042 |
| 0.1 | 0.4 | 0.5 | 0.008697689 | 0.9147886 |
| 0.1 | 0.6 | 0.6 | -0.059749 | 0.9656408 |
| 0.2 | 0.3 | 0.1 | 0.9696736 | 3.511172 |
| 0.3 | 0.3 | 0.2 | 0.916672 | 2.373731 |
| 0.4 | 0.3 | 0.2 | 0.7812134 | 9.273318 |
| 0.8 | 0.5 | 0.6 | -0.2128008 | 1.200246 |
| 0.9 | 0.6 | 1 | -0.2271696 | 1.220157 |
| 1 | 1 | 1 | -0.2618595 | 1.30627 |
