# Supplementary material for: A Gull Alpha Power Weibull distribution with applications to real and simulated data
Source: PLoS One. 2020 Jun 12;15(6):e0233080. doi: 10.1371/journal.pone.0233080 (PMC7292407; doi:10.1371/journal.pone.0233080)
Supplement: S2 Table — (DOCX) [file pone.0233080.s002.docx]

**Table 2: Maximum likelihood estimates and their standard errors**

| Model | Mle | Standard error | -log(likelihood) |
| --- | --- | --- | --- |
| GAPW | 0.00590119 0.79751413 0.53355796 | 0.005280265  0.121546525  0.046158047 | 409.9908 |
| W.E | 3.95810505 0.01796843 0.85819193 | 1.214089581  0.004666546  0.059280045 | 419.8998 |
| W | 0.09438292  1.04576466 | 0.01912624  0.06742473 | 414.0874 |
| Exp | 0.1067695 | 0.009436355 | 414.3419 |
| Rayleigh | 0.005079773 | 0.0004307331 | 491.2659 |
| AIFW | 0.1677404  0.1231948 | 0.02508775  0.01045528 | 451.0704 |
