# Supplementary material for: A Gull Alpha Power Weibull distribution with applications to real and simulated data
Source: PLoS One. 2020 Jun 12;15(6):e0233080. doi: 10.1371/journal.pone.0233080 (PMC7292407; doi:10.1371/journal.pone.0233080)
Supplement: S3 Table — (DOCX) [file pone.0233080.s003.docx]

**Table 3: Goodness of fit measures of the GAPW for bladder cancer data**

| Models | W | A | AIC | CAIC | BIC | HQIC |
| --- | --- | --- | --- | --- | --- | --- |
| GAPW | 0.02533431 | 0.1608187 | 825.9815 | 826.1751 | 834.5376 | 829.4579 |
| W.E | 0.2145276 | 1.282891 | 845.7996 | 845.9931 | 854.3557 | 849.276 |
| W | 0.1308177 | 0.7832353 | 832.1747 | 832.2707 | 837.8788 | 834.4923 |
| Exp | 0.1192893 | 0.7159703 | 830.6838 | 830.7155 | 833.5358 | 831.8426 |
| Rayleigh | 0.4669078 | 2.732901 | 984.5318 | 984.5635 | 987.3838 | 985.6906 |
| AIFW | 0.5735798 | 3.457475 | 906.1409 | 906.2369 | 911.8449 | 908.4585 |
