# Supplementary material for: A Gull Alpha Power Weibull distribution with applications to real and simulated data
Source: PLoS One. 2020 Jun 12;15(6):e0233080. doi: 10.1371/journal.pone.0233080 (PMC7292407; doi:10.1371/journal.pone.0233080)
Supplement: S4 Table — (DOCX) [file pone.0233080.s004.docx]

**Table 4: Maximum likelihood estimates and their standard errors**

| Model | Mle | Standard error | -log(likelihood) |
| --- | --- | --- | --- |
| GAPW | 0.004584114 0.540116500 0.679696285 | 0.007821438 0.164012222 0.088528859 | 317.4891 |
| W.E | 3.97641055 0.02509669 1.24349114 | 4.16205665 0.01545783 0.15095850 | 320.9662 |
| W | 0.02971531 1.46144250 | 0.008598382 0.102215074 | 318.745 |
| Exp | 0.1012424 | 0.01012326 | 329.0209 |
| Rayleigh | 0.006676124 | 0.0006522215 | 329.2404 |
| AIFW | 1.6423171 0.1153154 | 0.17122546 0.01167334 | 330.7856 |
