# Supplementary material for: A Gull Alpha Power Weibull distribution with applications to real and simulated data
Source: PLoS One. 2020 Jun 12;15(6):e0233080. doi: 10.1371/journal.pone.0233080 (PMC7292407; doi:10.1371/journal.pone.0233080)
Supplement: S5 Table — (DOCX) [file pone.0233080.s005.docx]

**Table 5: Goodness of fit measures of the GAPW for Bank Customers data**

| Model | W | A | AIC | CAIC | BIC | HQIC |
| --- | --- | --- | --- | --- | --- | --- |
| GAPW | 0.01939983 | 0.1355293 | 640.9783 | 641.2283 | 648.7938 | 644.1413 |
| W.E | 0.112468 | 0.7070021 | 647.9323 | 648.1823 | 655.7479 | 651.0954 |
| W | 0.06265989 | 0.3945532 | 641.4899 | 641.6136 | 646.7003 | 643.5986 |
| Exp | 0.02703835 | 0.1790246 | 660.0418 | 660.0826 | 662.6469 | 661.0961 |
| Rayleigh | 0.1265804 | 0.7863305 | 660.4807 | 660.5216 | 663.0859 | 661.5351 |
| AIFW | 0.1703407 | 1.219407 | 665.5711 | 665.6948 | 670.7815 | 667.6798 |
