# Supplementary material for: A Gull Alpha Power Weibull distribution with applications to real and simulated data
Source: PLoS One. 2020 Jun 12;15(6):e0233080. doi: 10.1371/journal.pone.0233080 (PMC7292407; doi:10.1371/journal.pone.0233080)
Supplement: S6 Table — (DOCX) [file pone.0233080.s006.docx]

**Table 6: Maximum likelihood Estimates and their standard errors**

| Actual values     | n | ML Estimate $\alpha$ $\beta$    | | | Standard deviations     | | |
| --- | --- | --- | --- | --- | --- | --- | --- |
| 0.009, 0.007, 2.5 | 30 | 52.356569 | 23.944706 | -1.425906 | 9.2277595 | 7.2776554 | 0.1274975 |
|  | 50 | 50.95129 | 24.95536 | -1.43942 | 6.9704413 | 6.0708245 | 0.1017499 |
|  | 60 | 50.664975 | 26.246471 | -1.466574 | 6.31080601 | 5.72063698 | 0.09179273 |
| 0.002, 0.03,  2.4 | 30 | 48.053781 | 30.374984 | 2.557561 | 8.5074528 | 11.2457262 | 0.2579958 |
|  | 40 | 54.455222 | 38.392647 | -2.634047 | 8.3220182 | 10.0698906 | 0.1794368 |
|  | 50 | 50.95129 | 24.95536 | -1.43942 | 6.9704413 | 6.0708245 | 0.1017499 |
| 0.004, 0.04,  2.4 | 15 | 43.061076 | 27.780158 | -2.690999 | 10.4921960 | 12.5321045 | 0.3456288 |
|  | 30 | 44.815687 | 32.610212 | -2.716042 | 7.8711814 | 11.2785370 | 0.2546263 |
|  | 60 | 58.716823 | 26.033329 | -2.651458 | 7.4268484 | 6.0626125 | 0.1738104 |
| 0.009, 0.05  3.5 | 30 | 74.154700 | 38.831954 | -4.727522 | 13.5628738 | 14.8703835 | 0.4515338 |
|  | 45 | 77.262356 | 35.034728 | -4.703549 | 11.5691089 | 10.7248896 | 0.3635661 |
|  | 60 | 63.771730 | 37.754394 | -4.728007 | 8.1322331 | 10.1954503 | 0.3217287 |
