# Supplementary material for: A Gull Alpha Power Weibull distribution with applications to real and simulated data
Source: PLoS One. 2020 Jun 12;15(6):e0233080. doi: 10.1371/journal.pone.0233080 (PMC7292407; doi:10.1371/journal.pone.0233080)
Supplement: S1 Data — (DOCX) [file pone.0233080.s007.docx]

**Data set 1: Remission time of Bladders cancer patients**

The data set consists of the remission time of 128 bladder cancer patients. The data set is taken from Aldeni and Famoye [29] with the values are as follows

0.080, 0.200, 0.400, 0.500, 0.510, 0.810, 0.900, 1.050, 1.190, 1.260, 1.350, 1.400, 1.460, 1.760, 2.020, 2.020, 2.070, 2.090, 2.230, 2.260, 2.460, 2.540, 2.620, 2.640, 2.690, 2.690, 2.750, 2.830, 2.870, 3.020, 3.250, 3.310, 3.360, 3.360, 3.480, 3.520, 3.570, 3.640, 3.700, 3.820, 3.880, 4.180, 4.230, 4.260, 4.330, 4.340, 4.400, 4.500, 4.510, 4.870, 4.980, 5.060, 5.090, 5.170, 5.320, 5.320, 5.340, 5.410, 5.410, 5.490, 5.620, 5.710, 5.850, 6.250, 6.540, 6.760, 6.930, 6.940, 6.970, 7.090, 7.260, 7.280, 7.320, 7.390, 7.590, 7.620, 7.630, 7.660, 7.870, 7.930, 8.260, 8.370, 8.530, 8.650, 8.660, 9.020, 9.220, 9.470, 9.740, 10.06, 10.34, 10.66, 10.75, 11.25, 11.64, 11.79, 11.98, 12.02, 12.03, 12.07, 12.63, 13.11, 13.29, 13.80, 14.24, 14.76, 14.77, 14.83, 15.96, 16.62, 17.12, 17.14, 17.36, 18.10, 19.13, 20.28, 21.73, 22.69, 23.63, 25.74, 25.82, 26.31, 32.15, 34.26, 36.66, 43.01, 46.12, 79.05.
