# Supplementary material for: A Gull Alpha Power Weibull distribution with applications to real and simulated data
Source: PLoS One. 2020 Jun 12;15(6):e0233080. doi: 10.1371/journal.pone.0233080 (PMC7292407; doi:10.1371/journal.pone.0233080)
Supplement: S2 Data — (DOCX) [file pone.0233080.s008.docx]

**Data set 2: Bank customers Data**

The data set waiting time of 100 bank customers is taken from Ghitany et al. [30]. The data set values are given below

0.8,0.8,1.3,1.5,1.8,1.9,1.9,2.1,2.6,2.7,2.9,3.1,3.2,3.3,3.5,3.6,4,4.1,4.2,4.2,4.3,4.3,4.4,4.4,4.6,4.7,4.7,4.8,4.9,4.9,5.0,5.3,5.5,5.7,5.7,6.1,6.2,6.2,6.2,6.3,6.7,6.9,7.1,7.1,7.1,7.1,7.4,7.6,7.7,8,8.2,8.6,8.6,8.6,8.8,8.8,8.9,8.9,9.5,9.6,9.7,9.8,10.7,10.9,11.0,11.0,11.1,11.2,11.2,11.5,11.9,12.4,12.5,12.9,13.0,13.1,13.3,13.6,13.7,13.9,14.1,15.4,15.4,17.3,17.3,18.1,18.2,18.4,18.9,19.0,19.9,20.6,21.3,21.4,21.9,23,27,31.6,33.1,38.5.
